# Effects of Nano-Silver Exposure on Oxidative Stress, Transcriptome, and Intestinal Microbiota of *Procambarus clarkii*

**DOI:** 10.3390/biology15010006

**Published:** 2025-12-19

**Authors:** Jian Li, Bin Qiu, Yitian Chen, Yanping Cai, Huiling Zhang, Xingfei Huang, Yude Wang, Shaojun Liu

**Affiliations:** Engineering Research Center of Polyploid Fish Reproduction and Breeding of the State Education Ministry, Hunan Normal University, Changsha 410081, China; 17871935018@163.com (J.L.); 15674994225@163.com (B.Q.); 18073715081@163.com (Y.C.); 15211162800@163.com (Y.C.); z1465402103@163.com (H.Z.); m19313071623@163.com (X.H.)

**Keywords:** *Procambarus clarkii*, nano-silver, oxidative stress, intestinal microbiota, transcriptome

## Abstract

There is limited understanding regarding the potential toxicity of nano-silver to crayfish. This study aims to evaluate the histopathological changes, oxidative stress, transcriptomics, and intestinal microbiota changes in different tissues of crayfish after exposure to nano-silver. The results showed that exposure to nano-silver caused pathological changes in the muscles, hepatopancreas, and gills of crayfish. Damage to muscular tissue progressively worsened with increasing concentrations of nano-silver, leading to a gradual widening of the gaps between muscle fibers. Nano-silver enlarged hepatopancreatic lumen and epithelial vacuolation, while the structure of the gills became disorganized, with severe damage to the gill membranes. The activities of peroxidase (CAT), superoxide dismutase (SOD), and glutathione reductase (GSH), as well as the content of malondialdehyde (MDA) in the muscles, hepatopancreas, and gills, were altered due to nano-silver exposure. Furthermore, along with the alteration of intestinal flora, there were alterations in the diversity of intestinal microbiota, an increase in the abundance of Bacteroides and Ca_Bacilloplasma, and a decrease in the abundance of Citrobacter. The abundance of harmful bacteria increased, causing intestinal inflammatory damage. Totals of 1549 and 1305 differently expressed genes (DEG) were found in the muscles and hepatopancreas, according to transcriptome analysis. Significantly affected pathways included the PPAR signaling pathway. These findings provide valuable insights into the use of nano-silver in the aquaculture of crayfish.

## Simple Summary

In this study, we evaluated the histopathological alterations, oxidative stress, transcriptomics, and intestinal microbiota changes in different tissue of red swamp crayfish (*Procambarus clarkii*) following exposure to nano-silver, which has been demonstrated to be an effective antibacterial agent widely used in water disinfection, including the improvement of water quality in aquaculture pond systems. The results indicate that nano-silver exposure adversely affects multiple biological processes in crayfish, suggesting a potential threat to its aquaculture.

## 1. Introduction

*Procambarus clarkii* belongs to *Arthropoda*, *Crustacea*, *Decapoda*, *Cambaridae*, and *Procambarus*. In 1929, crayfish were brought to the Nanjing in China. Due to the suitable conditions, crayfish have bred along the Yangtze River Basin. According to the “China Crayfish Industry Development Report (2025)”, the area for crayfish farming in China reached 20,333 square kilometers, and the output was 3.4476 million tons in 2024 [1], reflecting increases of 3.39% and 9.07%, respectively, compared to 2023. The output of crayfish farming accounts for 9.76% of the total freshwater aquaculture output in China, with the proportion increasing by 0.5 percentage points year-after-year. According to the “China Crayfish Industry Development Report (2025)” [1], crayfish ranks fourth among freshwater aquaculture varieties in China (the top three are grass carp, silver carp, and bighead carp). The development of the crayfish industry is rapid.

During their growth and development, they are prone to infection by various pathogens, including parasites, viruses, and bacteria [2]. This not only affects the growth performance of crayfish but may even lead to a high mortality rate, seriously affecting the healthy development of the crayfish-related breeding industry. In recent years, the research of crayfish mainly focused on disease prevention. Considering the intricate of pathogenicity affecting crayfish, this increases their susceptibility to diseases and leads to a significant mortality rate during the breeding process [3,4]; the development of ecologically friendly or pollution-free therapeutic agents is vitally important.

Nano-silver is a kind of metallic silver element with a particle size of 1–100 nm [5], which has strong antibacterial and antiviral effects [6,7,8]. Nanomaterials have also been used for disease prevention in white shrimp farming [9]. Nano-silver is often used as an antibacterial agent, exhibiting notable bactericidal effects and contributing to improved water quality [10,11]. Oxidative stress and inflammatory responses are caused by the presence of organisms treated with nano-silver at high concentrations [12]. However, high concentrations of nano-silver may affect crustacean aquatic animals, which attach to the shell and appendages, thereby impairing their biological functions [13]. Nano-silver causes biological accumulation in aquatic organisms and affects ecology and food safety [14,15]. In the toxicity test of nano-silver on zebrafish embryos, death and developmental abnormalities occurred [16,17]. Nano-silver exhibited tissue-specific accumulation in zebrafish, leading to morphological changes in gills and other tissues [18]. In aquaculture, the method of using nano-silver liquid is usually to sprinkle it into ponds for water disinfection. Crayfish are susceptible to different aquatic environment and chemical exposure, making them an ideal model organism for environmental studies [19]. Crayfish thrive in freshwater environments, which are characterized by high fecundity and rapid growth rates [20,21].

The main research directions of crayfish include nutrition physiology, metabolism, and environmental stress [22,23]. The toxicity of harmful substances to aquatic organisms leads to oxidative stress, accumulating a large number of reactive oxygen species and causing damage to organisms [24]. Organisms activate the detoxification mechanism of their antioxidant systems to counter oxidative stress [25]. Under ammonia nitrogen stress, superoxide dismutase (SOD) activity in the hepatopancreas of shrimp significantly decreased, while malondialdehyde (MDA) levels were significantly elevated [26]. Acute nitrite stress can alter the serum physiological status of crayfish, inducing oxidative stress and causing damage to the gills. Aluminum exposure affects the immunity of crayfish, significantly inducing oxidative stress and pathological histological changes in the intestine [27]. Muscle tissue maintains normal energy metabolism function [28]; once damaged, its normal growth and development are affected. The edible part of crayfish is mainly muscle, and it accounts for the largest part of the whole mass [29]. Changes in enzyme activity in muscle tissue can reflect the degree of muscle injury [30]. It is important to study the effects of nano-silver on the muscle tissue and growth or feeding of crayfish.

In order to investigate the potential risks of nano-silver to crayfish, we conducted a comprehensive assessment through histological observation of oxidative stress, intestinal microbiota, and transcriptome analysis. The changes in MDA, SOD, CAT, and GST in crayfish were studied. Histopathological analysis of the effects of silver nanoparticles on muscles, hepatopancreas, and gills was performed. We simultaneously analyzed the transcriptome and the intestinal microbiota, identified key pathways, and examined the changes in the microbiota to reveal the regulatory mechanisms of crayfish under nano-silver stress at the molecular level and the rational use of nano-silver in the actual breeding process.

## 2. Materials and Methods

### 2.1. Experimental Animals and Conditions

Healthy crayfish were obtained from Hunan Normal University, and experiments were conducted here. A total of 450 male crayfish individuals were randomly distributed into five groups, with three replicates. Each group contained 30 individuals (10 ± 0.3 g). The crayfish were placed in 15 plastic boxes (size: 120 cm × 60 cm × 40 cm) under experiment conditions (dissolved oxygen at 7.1 mg/L, pH 7.5 ± 0.3, ammonia nitrogen < 0.2 mg/L, nitrite < 0.005 mg/L, and temperature at 24 ± 2 °C), each containing 60 L of water to soak the crayfish. All the crayfish were fed with commercial pellet feed twice a day. After 15 days of acclimatization, a formal experiment was conducted. Every night, we cleaned the residual feed. The crayfish were randomly assigned to five groups and exposed to nano-silver concentrations of 0, 15, 30, 45, and 60 mg/L, respectively. Status of the animals was checked every two hours, and any dead individuals were counted and immediately removed. The exposure solution was manually renewed once a day. At 24, 48, 72, and 96 h of exposure, three crayfish were randomly selected. Following hypothermia anesthesia, a small amount of muscle tissue was collected from each crayfish and subsequently frozen at −80 °C. The mortality rates of crayfish were recorded as 0%, 10%, 10%, 13%, and 23% at 0, 15, 30, 45, and 60 mg/L of nano-silver after 72 h, respectively.

### 2.2. Chemicals and Reagents

The nano-silver (particle size 1–100 nm) was provided by Beijing Jingpeng Runze Industrial Co., Ltd. (Beijing, China) and was stored at 4 °C in a dark place. Kits for measuring SOD, GST, CAT, and MDA were purchased from Hubei Pumoke Biotechnology Co., Ltd. (Wuhan, China).

### 2.3. Enzyme Activity Determination

Crayfish was exposed to nano-silver for 72 h. MDA [31] content and the activities of CAT [32], GSH [33], and SOD [34] in muscle tissue were measured using corresponding kits. All kits were obtained from Hubei Pumoke Biotechnology Co., Ltd., and all procedures were conducted according to the manufacturer’s instructions.

### 2.4. Histological Observation

Crayfish were exposed to nano-silver at concentrations of 0, 15, 30, 45, and 60 mg/L for 72 h. Muscle, hepatopancreas, and gill tissue were fixed in 4% paraformaldehyde solution for 24 h and then subjected to histopathological examination. These steps involve dehydration, embedding, section, staining with hematoxylin and eosin (H&E), and observing the section with a microscope (Leica, Wetzlar, Germany).

### 2.5. Analysis of Intestinal Microbiota

The microbial genomic DNA was extracted from the intestinal contents of the crayfish. The V3-V4 region of the bacterial 16S rRNA gene was amplified using the primers 341F: CCTACGGGNGGCWGCAG and 806R: GGACTACHVGGGTWTCTAAT. The PCR amplification products were gel-purified and quantified using a QuantiFluor^TM^ fluorometer (Promega Corporation, Madison, WI, USA). The purified amplification products were ligated with sequencing adapters, and libraries were constructed. Then, sequencing was performed on the Illumina PE250 platform. DADA2 was used to concatenate, quality control, and filter the data. The bacterial communities were analyzed at the phylum, family, and genus levels to identify differences. The difference in microbiota was examined by the Effect Size Measurement (LEfSe) in conjunction with discriminant analysis (LDA). Data processing and bioinformatics analysis were conducted using the Illumina HiSeq 2500 platform (Guangzhou Kigen Bio-Technology Co., Ltd., Guangzhou, China).

### 2.6. Transcriptome Sequencing Analysis

Total RNA was extracted from the collected muscle using the Invitrogen Trizol kit (Thermo Fisher Scientific, Waltham, MA, USA) and detected by 1% agarose gel electrophoresis. RNA purity and integrity were assessed using a NanoDrop 2000 (Thermo Fisher Scientific, USA) and an Agilent 2100 Bioanalyzer (Agilent, Santa Clara, CA, USA). The construction of the Illumina library was conducted by Biomarker Technologies (Beijing, China), and the quality control of the raw data was performed using fastp (v0.18.0). Quality-controlled sequences were aligned to reference genomes using Hisat2 (v2.2.1) to generate alignment data. Read count data were normalized using TMM (v4.0.16), and differential expression analysis was conducted using DEGseq (v1.20.0). DEG were identified using the following criteria: q < 0.05 and |Fold Change| > 2. Gene Ontology (GO); Kyoto Encyclopedia of Genes and Genomes (KEGG) enrichment analyses were performed to determine DEG functions and associated pathways (q < 0.05).

### 2.7. Quantitative RT-PCR Assay

The total RNA extracted was reverse-transcribed into cDNA by the one-step method (Takara, RR047A, Kyoto, Japan). Primers were designed using Primer Premier 5.0 software and synthesized by Beijing Qingke Biotechnology Co., Ltd. (Beijing, China), with 18S rRNA serving as the internal reference gene for qRT-PCR. The primers for internal reference genes are listed in Table 1. The relative expression levels were validated using the 2^−△△Ct^ method [35].

### 2.8. Statistical Analysis

The experimental data were analyzed using SPSS 20.0 software. Data are presented as mean ± SD. Differences between groups were evaluated using one-way ANOVA followed by LSD test. *p*-value < 0.05 was considered statistically significant.

## 3. Results

### 3.1. Histological Observation

**Figure 1:**
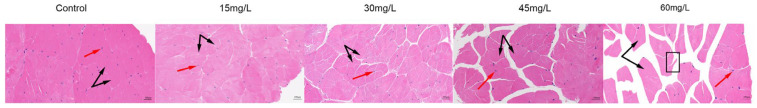
Histological changes in crayfish muscles after exposure to nano-silver for 72 h. The black arrows in the figure indicate the muscle fibers, the red arrows represent the cell nuclei, and the boxes show the broken muscle fibers.

The changes in muscle tissue sections of crayfish after contact with nano-silver at 72 h exposure are shown in Figure 1. In the control group, the overall structure of the muscle tissue was complete, the muscle fibers were closely arranged, the gap between each other was small, and the cell nuclei were evenly distributed. Compared with the control group, the degree of damage to crayfish muscle tissue was different from that of nano-silver at 72 h exposure. As the nano-silver concentration continued to increase, the gap between muscle fibers gradually widened. After 72 h of contact with 60 mg/L nano-silver, the muscle structure was incomplete and damaged, the muscle fibers were fractured and fragmented, and the dissolution of a large number of nuclei was observed.

**Figure 2:**
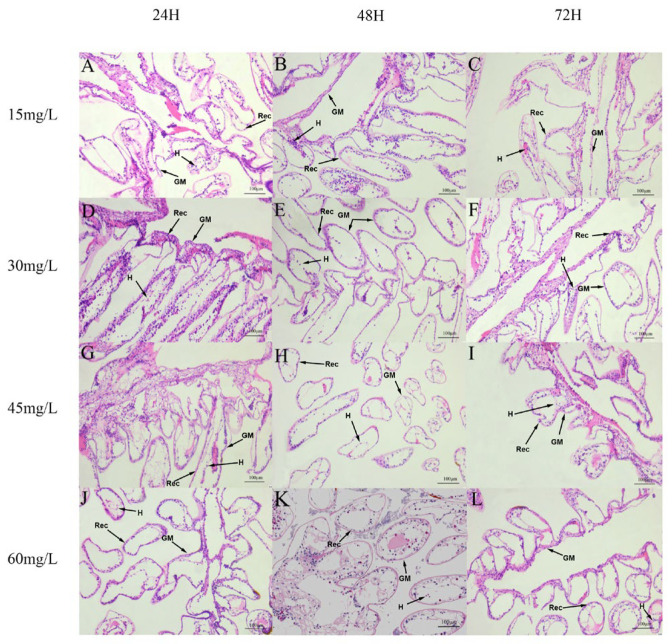
Effects of nano-silver exposure on the gill structure of crayfish. (**A**–**C**) Total of 15 mg/L nano-silver for 24 h, 48 h, and 72 h. (**D**–**F**) Total of 30 mg/L nano-silver for 24 h, 48 h, and 72 h. (**G**–**I**) Total of 45 mg/L nano-silver for 24 h, 48 h, and 72 h. (**J**–**L**) Total of 60 mg/L nano-silver for 24 h, 48 h, and 72 h. Rec, respiratory epithelium cells; H, hemocytes; GM, gill membranes.

At low concentrations, the gill membrane was relatively intact, the structure of respiratory epithelium cells (REC) was clear, and the number of hemocytes was relatively high (Figure 2A–C). As the concentration increased, the looseness of gill filaments increased (Figure 2D–I). After 72 h of exposure to 60 mg/L nano-silver, most of the REC were loosely arranged and separated, the number of hemocytes decreased, the gill membrane was damaged, and the normal structure of the gill was lost (Figure 2J–L).

**Figure 3:**
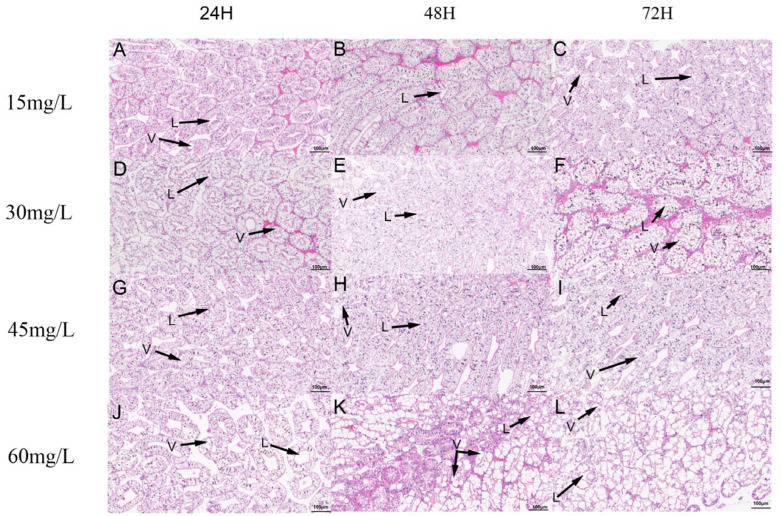
Microscopic photographs of hepatopancreas histological. (**A**–**C**) Total of 15 mg/L for 24 h, 48 h, and 72 h. (**D**–**F**) Total of 30 mg/L nano-silver for 24 h, 48 h, and 72 h. (**G**–**I**) Total of 45 mg/L nano-silver for 24 h, 48 h, and 72 h. (**J**–**L**) Total of 60 mg/L nano-silver for 24 h, 48 h, and 72 h. L: lumen; V: vacuolization.

At low concentrations, the hepatopancreas showed well-organized structures, with the tubular cavities presenting an asterisk-like appearance (Figure 3A–F). As the concentration of nano-silver exposure increased, the hepatopancreas of the crayfish was affected, including the expansion of the lumen, degeneration of the tubules, and vacuolation of the epithelium (Figure 3G–I). All the hepatopancreas treated at 45 mg/L showed degenerated tubules, and all the treated samples showed expansion of the lumen (Figure 3J–L). Specifically, the hepatopancreas exposed to nano-silver for 72 h exhibited obvious epithelial vacuolation. All the nano-silver-treated hepatopancreas showed expansion of the lumen. 

### 3.2. Oxidative Stress and Antioxidant Enzymes

**Figure 4:**
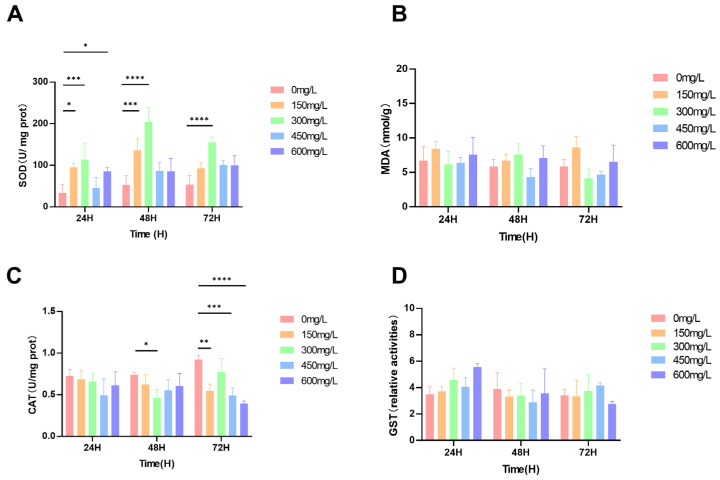
(**A**) SOD, (**B**) MDA, (**C**) CAT, and (**D**) GST content and activity levels. The values of all parameters are expressed as mean ± SD, *n* = 3. * (*p* < 0.05), ** (*p* < 0.01), *** (*p* < 0.001), and **** (*p* < 0.001).

As shown in Figure 2A, the SOD activity of all treated crayfish increased to varying degrees. Compared with the control group, SOD activity significantly increased, reaching the highest levels after 24, 48, and 72 h of exposure to 30 mg/L nano-silver, with increases of 235%, 247%, and 191%, respectively (*p* < 0.01) (Figure 4A). No significant differences in MDA content were observed compared to the control group (*p* > 0.05) (Figure 4B). CAT activity was significantly inhibited after 48 h of exposure to 30 mg/L nano-silver. After 72 h of exposure to 15, 45, and 60 mg/L nano-silver, CAT activity decreased by 40.5%, 46.8%, and 57.4%, respectively. After 48 h of exposure to 30 mg/L nano-silver, CAT activity decreased by 37.9% (Figure 4C). GST activity was significantly increased after 24 h of exposure to 60 mg/L nano-silver, but no significant changes were observed under other conditions (*p* > 0.05) compared to the control group (Figure 4D).

**Figure 5:**
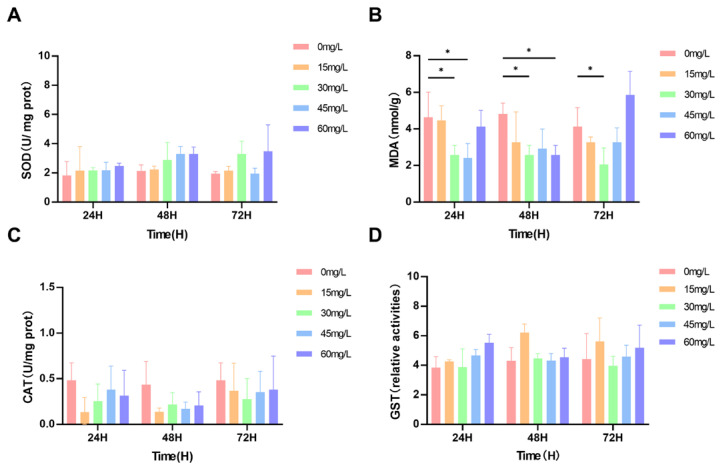
(**A**) SOD, (**B**) MDA, (**C**) CAT, and (**D**) GST content and activity levels. The values of all parameters are expressed as mean ± SD, *n* = 3. * (*p* < 0.05).

SOD activity of all crayfish treated with nano-silver increased (*p* > 0.05). SOD activity decreased significantly at 30 mg/L after 72 h (*p* < 0.05) (Figure 5A). When the gills were exposed to nano-silver for 24 h and 48 h, MDA content decreased significantly at 30 and 45 mg/L compared to 30 mg/L at 72 h (*p* < 0.05) (Figure 5B). CAT activity of all crayfish treated with nano-silver decreased (*p* > 0.05) (Figure 5C). Except for the 30 mg/L at 72 h which decreased, all GST activity increased (*p* > 0.05) (Figure 5D).

**Figure 6:**
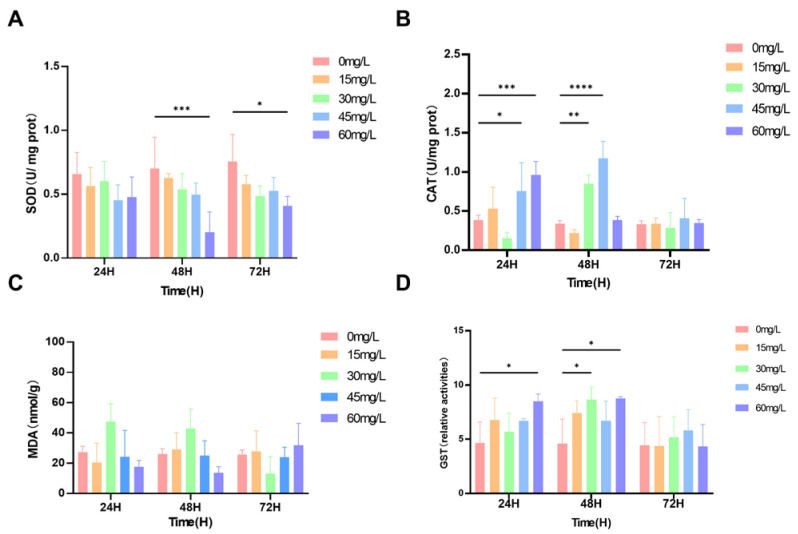
(**A**) SOD, (**B**) MDA, (**C**) CAT, and (**D**) GST content and activity levels. The values of all parameters are expressed as mean ± SD, *n* = 3. * (*p* < 0.05), ** (*p* < 0.01), *** (*p* < 0.001), and **** (*p* < 0.001).

SOD activity significantly decreased at the 48 h and 72 h exposure times at a concentration of 60 mg/L (Figure 6A). Similarly, when the crayfish were exposed to nano-silver at concentrations of 45 and 60 mg/L for 24 h and 48 h, CAT activity was observed to increase significantly (Figure 6B). Compared with the control sample, MDA content in the hepatopancreas did not show a significant difference (*p* > 0.05) after the hepatopancreas was exposed to all test concentrations of nano-silver for 72 h (Figure 6C). Exposure to 60 mg/L nano-silver for 24 h and 48 h led to a significant increase in GST activity, and 30 mg/L nano-silver for 48 h also showed a significant increase (*p* < 0.05) (Figure 6D).

### 3.3. Analysis of Intestinal Microbiota

**Figure 7:**
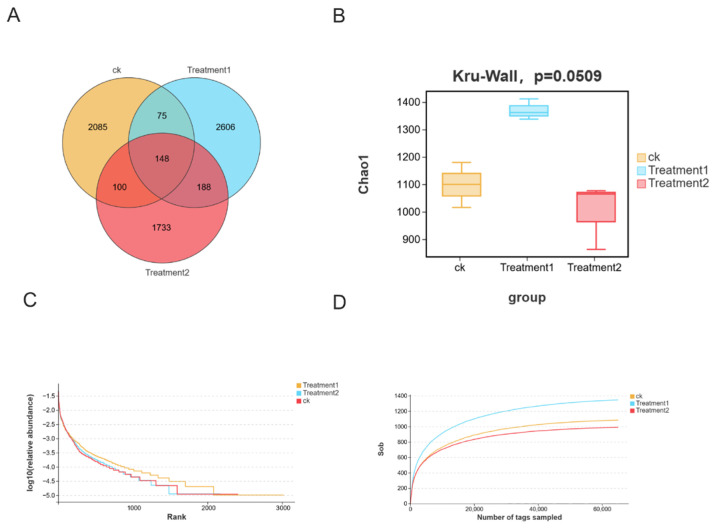
(**A**) Venn diagram shows the number of shared and unique OTUs; (**B**) α-diversity index (Chao1); (**C**) rarefaction curve; (**D**) rank-abundance curve. All data are presented as mean ± SD (*n* = 3).

Exposure to nano-silver altered the intestinal microbiota. We conducted an analysis of the intestinal microflora after exposing crayfish to 0, 30, and 60 mg/L nano-silver for 72 h. The Venn diagram revealed a total of 6935 OTUs. Among them, 148 OTUs were common to all three groups, and 2085 were specific to the control group (Figure 7A). The α and β diversity of the intestinal microbiota in the crayfish exposed to nano-silver were analyzed. The Chao1 index showed a significant difference in treatment group 1 (Figure 7B). The end point of each sample was generally flat on the sparse curve, indicating that the sequencing quantity was sufficient (Figure 7C,D).

#### Diversity of Intestinal Microbiota

**Figure 8:**
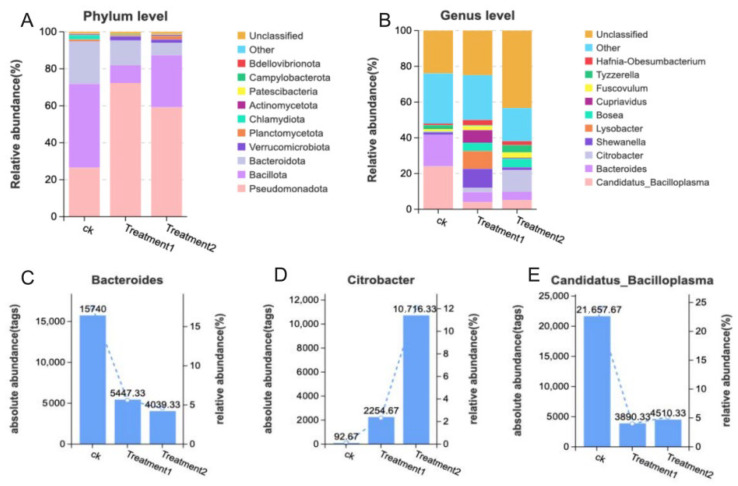
Comprehensive exposure affected the structure and abundance of the intestinal microbiota of the crayfish. (**A**) Histogram of the intestinal microbial components at the phylum level. (**B**) Histogram of the intestinal microbial components at the genus level. (**C**–**E**) Histogram of the relative abundances of Bacteroides, Citrobacter, and Candidatus_Bacilloplasma.

At the phylum level, the dominant intestinal microorganisms are Bacteroida, Bacillota, and Pseudomonadota (Figure 8A). At the genus level, the main intestinal microorganisms are Bacteroides, Candidatus_Bacilloplasma, and Citrobacter (Figure 8B). The histograms of the relative abundances of the three bacteria are demonstrated in (Figure 8C–E).

**Figure 9:**
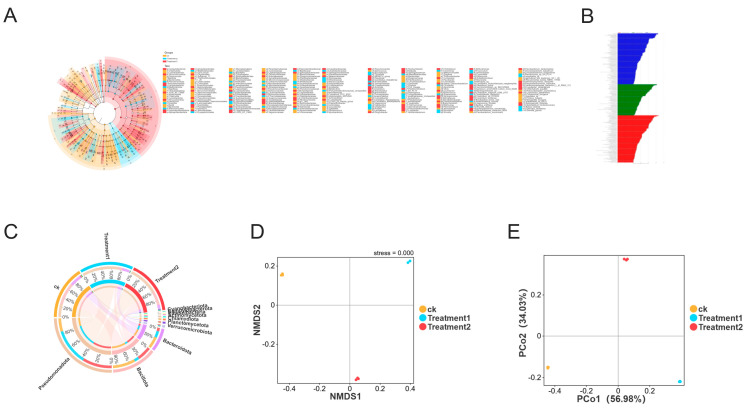
The diversity of the intestinal microbial community of Macrobrachium rosenbergii treated with nano-silver. (**A**) Classification diagram of LEfSe. (**B**) LDA score (threshold of 3). (**C**) Circos diagram of the phylum-level intestinal microbial community. (**D**,**E**) β-diversity represented at the OTU level using NMDS and PCoA.

The imbalance of the intestinal microbiota may cause abnormal immune responses and increased intestinal permeability, allowing harmful bacteria to pass through the intestinal wall and causing the death of the crayfish. To identify the bacteria that may be altered by nano-silver and affect the disruption of the intestinal microbiota, a high-dimensional class comparison analysis using the linear discriminant analysis effect size (LEfSe) was conducted. This analysis showed significant changes in the dominance of the microbial community (Figure 9A,B). Among them, the levels of Pseudomonadota and planctomycetota increased, which were the key bacterial types that mediated the dysbiosis of the intestinal microbiota in the nano-silver group. At the phylum level (Figure 9C), the richness of Bacillota in the control group decreased from 54.62% to 11.46% in treatment group 1 and 33.92% in treatment group 2, while the richness of Pseudomonadota increased from 16.75% to 45.83% in treatment group 1 and 37.43% in treatment group 2. In addition, the β-diversity analysis (NMDS and PCoA analysis) showed that the overall structural changes in the intestinal microbiota caused by nano-silver led to a change in the intestinal microbiota structure of the crayfish (Figure 9D,E).

### 3.4. Transcriptome Analysis

**Figure 10:**
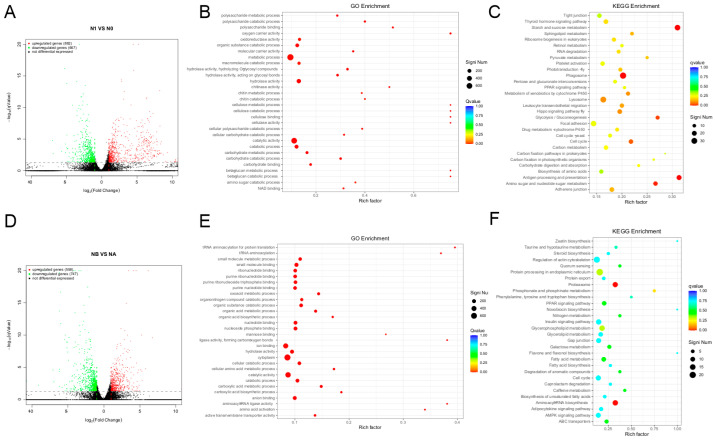
Functional annotation analysis. (**A**,**D**) Volcano maps show common and specific DEGs; red dots represent up-regulated genes and green dots represent down-regulated genes. (**B**,**E**) GO enrichment graphs of DEGs representing the top 30 GO terms in enrichment analysis. (**C**,**F**) KEGG-enriched bubble plots of DEGs, showing the first 30 pathways of enriched DEGs in KEGG analysis.

Crayfish were exposed to nano-silver at concentrations of 0 and 60 mg/L for 72 h. Transcriptome analysis of the muscles and the hepatopancreas revealed 1549 DEGs (882 genes up-regulated and 667 down-regulated) and 1305 DEGs (558 genes up-regulated and 747 down-regulated) (Figure 10A,D). In GO enrichment analysis, the main enrichments were found in catalytic activity, catabolic process, and hydrolase activity (Figure 10B,C,E). KEGG pathway enrichment analysis was conducted, and the main enrichment was in the PPAR signaling pathway (Figure 10C,F). This indicates that contact with nano-silver may affect the immune and metabolic processes of lobsters. Nano-silver significantly influenced the immune response-related pathways of the crayfish, including the PPAR signaling pathway.

### 3.5. Verification of qRT-PCR Results for Transcriptome Data

**Figure 11:**
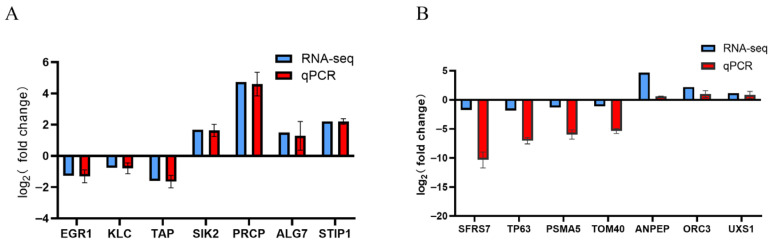
Verification of gene expression patterns of RNA-seq by qRT-PCR. (**A**) Muscle transcriptome. (**B**) hepatopancreas transcriptome.

To verify transcriptome results, seven DEGs from the nano-silver treatment group were randomly selected for qRT-PCR analysis (Figure 11A,B). The results confirm that RNA-seq data are accurate.

## 4. Discussion

This study investigates the response of crayfish to nano-silver. Through histological observations, analysis of oxidative stress, and transcriptome analysis, we elucidate the toxic mechanism of nano-silver-induced muscle injury in crayfish. MDA reflects the degree of lipids oxidation organisms and serves as an indicator of oxidative harm [36,37]. Antioxidant enzymes can play a crucial role in eliminating reactive oxygen radicals and safeguarding organisms from oxidative harm [38]. GST is an antioxidant that protects cells. It is used to eliminate free radicals and other oxidants [39]. CAT and SOD are the first line of defense against oxidative stress [40]. SOD is a key enzyme for eliminating reactive oxygen species (ROS), and excessive ROS can cause damage to cells [41]. Based on the changes in these oxidative stress markers, it can be concluded that nano-silver exposure induced oxidative stress responses in the crayfish.

Furthermore, the damage caused by nano-silver to tissues was studied through histopathological observation. In this study, nano-silver at 60 mg/L induced more pronounced damage. The observed changes included a gradual increase in muscle fiber gaps, the appearance of structural imperfections, broken muscle fibers, as well as dissolved and reduced nuclei. Nano-silver caused considerable tissue damage to muscle. When exposed to Cyhalofop-butyl and pyribenzoxim, crayfish muscle tissue also undergoes structural changes [30,42]. These results indicated that nano-silver might damage the muscle tissue of crayfish. The hepatopancreas plays significant roles in immune response and metabolism. Various stress treatments can damage the hepatopancreas of crayfish [43,44,45,46]. Our research observed that exposure to nano-silver resulted in hepatopancreas damage in crayfish, evident from the gradual dilation of the lumen and the formation of vacuoles. Additionally, gills are essential respiratory organs for aquatic organisms. When harmful substances in the water reach a certain concentration, they can damage the structure of the gill tissue, reducing respiratory capacity and leading to hypoxia [26,47]. In this experiment, we noted that a significant number of respiratory epithelial cells were arranged loosely, the blood cell count had decreased, the gill membrane was compromised, and the normal structure was disrupted, further affecting the gills’ physiological functions.

Shrimp grow and develop by absorbing nutrients through the intestine, so a stable intestinal microbiota is crucial for the survival of crayfish [48]. We further explored whether there was an impact on the intestinal microbiota, which plays a very critical role in maintaining the health of organisms [49]. Changes in the intestinal microbiota may be related to intestinal diseases, infections, and dysbiosis [50,51]. In this study, the intestinal microbial community of the crayfish exposed to nano-silver changed. Further analysis revealed that at the phylum level, the dominant intestinal microorganisms were Bacteroida, Bacillota, and Pseudomonadota. At the genus level, the main intestinal microorganisms were Bacteroides, Candidatus_Bacilloplasma, and Citrobacter. Pseudomonadota plays a key role in maintaining the stability of the intestinal microbiota [52], which is a major indicator of intestinal health. Mice with colitis have dysbiosis, characterized by lower microbial diversity and significantly elevated levels of Pseudomonadota [53]. After Maxing Shigan Decoction (MXSGD) treatment for pneumonia in mice, planctomycetota decreased, and MXSGD can regulate the intestinal microbiota and play a role in the treatment of pneumonia [54]. In this experiment, the increase in Pseudomonadota and planctomycetota levels in the nano-silver group was the key bacterial type mediating the dysbiosis of the intestinal microbiota. At the phylum level, the abundance of Bacillota in the control group (54.62%) decreased to 11.46% in treatment group 1 and 33.92% in treatment group 2, while the abundance of Pseudomonadota increased from 16.75% to 45.83% in treatment group 1 and 37.43% in treatment group 2. Additionally, β-diversity analysis (NMDS and PCoA analysis) showed that nano-silver caused overall structural changes in the intestinal microbiota, leading to alterations in the intestinal microbiota structure of crayfish. Overall, nano-silver exposure altered the diversity of the intestinal microbiota of crayfish, increasing the possibility of inflammation and microbial dysbiosis and raising the risk of morbidity.

Transcriptome analysis further elucidated the toxic mechanism of nano-silver on crayfish muscle. Transcriptome analysis of the muscles and the hepatopancreas revealed 1549 DEGs (882 genes up-regulated and 667 down-regulated) and 1305 DEGs (558 genes up-regulated and 747 down-regulated). In GO enrichment analysis, the main enrichments were found in catalytic activity, catabolic process, and hydrolase activity. KEGG analysis showed that the main enrichment was in the PPAR signaling pathway. Furthermore, muscles harbor various endogenous pro-oxidant and antioxidant systems [55]. When peroxidation overcomes antioxidant defenses, lipid peroxidation occurs, leading to muscle deterioration. Glucose metabolism in muscle critically influences dietary starch utilization [56]. Glucose is converted to pyruvate through glycolysis in the cytoplasm. Glycolysis produces a certain amount of energy from glucose and involves a complex series of enzyme reactions including HK, PK, and PFK [57]. Acetyl-CoA produced in the process is converted into fatty acids, which promote lipid accumulation and thus influence muscle composition. Anti-inflammatory factors mediate inflammatory immune responses through pathways such as PPAR signaling [58]. In this study, we found that nano-silver stress induced differential expression of genes in the PPAR signaling pathway of crayfish. Collectively, PPAR pathway up-regulation could enhance immune and metabolic capacity in crayfish.

In our experiment, the crayfish exposed to nano-silver showed tissue pathological damage, changes in antioxidant enzyme activity, intestinal microbial imbalance, and gene differences at the transcriptomic level. Overall, exposure to nano-silver led to intestinal microbial imbalance, oxidative stress, and immune system dysregulation in crayfish.

## 5. Conclusions

In conclusion, this study investigated the effects of nano-silver exposure on the tissue of crayfish. Nano-silver exposure induced significant damage to the tissue of crayfish and altered oxidative stress-related parameters. The diversity of the intestinal microbiota changes, and the abundance of harmful bacteria increases, caused intestinal inflammation and damage. Furthermore, transcriptome analysis revealed significantly enriched GO terms, KEGG pathways, and nano-silver-induced differentially expressed genes (DEGs), providing additional evidence. This study provides valuable insights into the toxicological effects and underlying mechanisms of nano-silver exposure in crayfish. The findings provide insights into the molecular-level regulatory mechanisms of crayfish in response to nano-silver, enhance the understanding of the effects of high-concentration nano-silver exposure on this species, and offer theoretical guidance for its aquaculture. However, nano-silver may pose potential threats to the growth and development of crayfish and food safety.

## Figures and Tables

**Table 1:** Primer sequences used for RT-qPCR analysis for related genes in this study.

Primer Name	Primer Sequences (5’-3’)
18S rRNA-F	GTCAGGTCATCACCATCGGCA
18S rRNA-R	CGGTCTCGTGAACACCAGCA
EGR1-F	GAGCTGACGCGACATATCCG
EGR1-F	GGCGAACTCCCACTCACTTTT
Klc-F	TGTGGATTTATTCCCTGACG
Klc-F	GTGCAAAGTTCGCAGACG
TAP-F	ATACATTGCTGATTTCACCCTC
TAP-F	ACCCAGCTTACTTGGTACTTGA
SIK2-F	TGCCTCGGATGGTTTAGT
SIK2-F	GCTGTCTGGTCGTTGTCTC
PCRP-F	TTCAAACCCTGGAGACC
PCRP-F	CTCGTCCTTATCATGTTTAGATTTA
ALG7-F	TAAACATAGTGGTGTCGGTAG
ALG7-F	TTTGTAGTGCGGTCTCGT
STIP1-F	CGCCGCAATCCCGAAGA
STIP1-F	TGGCTCCAGTCTGATGCACTCAT
TP63-F	CTCAAGACAAAGTCTGCCAAGT
TP63-R	CTGCGAAGTTCAGGTGAAGTAG
TOM40-F	CCAGGCACTATGGAAGAC
TOM40-R	CTGCTGAGTACCCACAAAC
PSMA5-F	GGCTCTAAAGATTGCGTTGA
PSMA5-R	GAAACCCTTCTCGGGGGTCACAGCT
SFRS7-F	CCTGAGGACCGTTGTTATG
SFRS7-R	GTATGATCTTCTCCTACGAGGT
ANPEP-F	GTCCTCCCATCCCATCA
ANPEP-R	AAAGGTCGTCCTGCTCTG
UXS1-F	AGACTGAGGCTTACTGGG
UXS1-R	ATACGTGCTACTCTTACTTCC
ORC3-F	TAAAGTGCTGGCAAATGG
ORC3-R	AAACACTGCATCGTGGTCT

## Data Availability

The data from the study are available from the corresponding authors upon reasonable request.

## Abbreviations

The following abbreviations are used in this manuscript:

CAT	catalase
SOD	superoxidedismutase
GST	glutathione-S-transferase
MDA	malondialdehyde
PPAR	peroxisome proliferators-activated receptors
